# Predicting spiking activity from scalp EEG

**DOI:** 10.1088/1741-2552/ae2541

**Published:** 2025-12-19

**Authors:** Dixit Sharma, Bart Krekelberg

**Affiliations:** 1 Center for Molecular and Behavioral Neuroscience, Rutgers University—Newark, Newark, NJ 07102, United States of America; 2 Graduate Program in Neuroscience, Rutgers University—Newark, Newark, NJ 07102, United States of America

**Keywords:** spiking activity, EEG, nonhuman primates, primary visual cortex, steady-state visual evoked potential, flickering visual stimulus

## Abstract

*Objective.* Despite decades of electroencephalography (EEG) research, the relationship between EEG and underlying spiking dynamics remains unclear. This limits our ability to infer neural dynamics reflected in intracranial signals from EEG, a critical step to bridge electrophysiological findings across species and to develop non-invasive brain–machine interfaces (BMIs). In this study, we aimed to estimate spiking activity in the visual cortex using non-invasive scalp EEG. *Approach*. We recorded spiking activity from a 32-channel floating microarray permanently implanted in parafoveal V1 and scalp-EEG in a male macaque monkey. While the animal fixated, the screen flickered at different temporal frequencies to induce steady-state visual evoked potentials. We analyzed the relationship between the V1 multi-unit spiking activity envelope (MUAe) and EEG frequency bands to predict MUAe at each time point from EEG. We extracted instantaneous spectrotemporal features of the EEG signal, including phase, amplitude, and phase-amplitude coupling of its frequency bands. *Main results*. Although the relationship between these spectrotemporal features and the V1 MUAe was complex and frequency-dependent, they were reliably predictive of the MUAe. Specifically, in a linear regression predicting MUAe from EEG, each EEG feature (phase, amplitude, coupling) contributed to model predictions. In addition, we found that MUAe predictions were better in shallow than deep cortical layers, and that the phase of stimulus frequency further improved MUAe predictions. *Significance.* Our study shows that a comprehensive account of spectrotemporal features of non-invasive EEG provides information on underlying spiking activity beyond what is available when only the amplitude or phase of the EEG signal is considered. This demonstrates the richness of the EEG signal and its complex relationship with neural spiking activity and suggests that using more comprehensive spectrotemporal signatures could improve BMI applications.

## 1. Introduction

Electroencephalography (EEG) is a widely used non-invasive method for measuring neuronal activity in humans, valued for its cost-effectiveness, portability, and high temporal resolution. Despite its limited spatial resolution compared to imaging techniques like fMRI, EEG’s ability to capture neural dynamics at high spectrotemporal resolution has made it an essential tool for diagnosing neurological disorders (Fisher *et al* 2005, Jiao *et al* 2023), investigating the neural correlates of cognitive processes (Friston 2009, Biasiucci *et al* 2019), and controlling non-invasive brain-computer interfaces (Birbaumer *et al* 2008).

There is a broad consensus that EEG signals primarily originate from the synchronized electrical activity of cortical neurons, specifically the (excitatory or inhibitory) postsynaptic potentials of pyramidal neurons oriented perpendicular to the cortical surface (Nunez and Srinivasan 2006, Buzsáki *et al* 2012). Their parallel (open-field) arrangement allows for spatial summation of postsynaptic potentials, resulting in strong extracellular dipole fields measured as EEG signals. However, in some cases, neurons in close-field arrangements, such as stellate cells, can also contribute to EEG activity (Tenke *et al* 1993; see Nunez and Srinivasan 2006 for a detailed review). Biophysically realistic compartmental neuron models highlight that neuronal spiking activity may also be a significant contributor to EEG (Thio and Grill 2023), and synchrony across neurons makes spiking activity more detectable with EEG electrodes (Murakami and Okada 2006). Although the physics underlying EEG signals is well understood, research exploring how the spectrotemporal features of EEG relate to underlying cortical signals, such as spiking activity and local field potentials (LFPs), remains limited.

A long-term objective of linking EEG with underlying cortical signals measured invasively is to develop tools that can estimate time-precise local cortical dynamics using non-invasive EEG signals. However, signal averaging and the low signal-to-noise ratio (SNR) of EEG make this challenging. Although complete realization may not be feasible in the short term, incremental advancements can help bridge the gap between invasive and non-invasive signals, and thus between animal and human neurophysiology research. Moreover, a better understanding of EEG’s spectrotemporal features could improve clinical biomarkers and support the development of non-invasive brain–machine interfaces (BMIs) and closed-loop neuromodulation systems (Lu *et al* 2021).

One promising approach to narrowing the gap between invasive and non-invasive recordings is to record both signals simultaneously in animals engaged in cognitive tasks. A few studies have used this approach to better understand the relationship between cortical signals and EEG. For example, Whittingstall and Logothetis (2009) found that multi-unit spiking activity in macaque V1 was weakly correlated with bone-surface EEG amplitude (*r* = − 0.12), but showed a stronger relationship with EEG delta phase (2–4 Hz) and gamma power (30–100 Hz). This frequency-dependent relationship can be exploited to estimate V1 spiking activity (Whittingstall and Logothetis 2009). Synchronization among cortical signals also impacts EEG: Snyder *et al* (2015) reported a non-linear relationship between spike synchronization in V4 and EEG alpha-band power, with higher alpha power observed during both low and high spike synchronization compared to moderate levels. The relationship between EEG and cortical signals also depends upon the visual stimulus (Snyder and Smith 2015).

Recent efforts have explored estimating brain activity from scalp EEG in humans, particularly in patients with epilepsy. Yamin *et al* (2023) demonstrated modest predictability of the average firing rates of neurons in the medial temporal lobe during sleep and wakefulness. Similarly, Subramanian *et al* (2025) found that intracranial spectral activity measured across multiple regions could be estimated from surface EEG, with accuracy decreasing with depth and frequency. These findings highlight the potential of statistical models that bridge the gap between invasive and non-invasive neural recordings, paving the way for non-invasive monitoring of neural activity in both clinical and research settings.

With the high-level goal of estimating cortical dynamics non-invasively, we build on previous studies to develop a comprehensive framework that enables time-precise spike dynamics estimation in macaque V1 using non-invasive EEG. Our approach incorporates two advancements over prior studies. First, to overcome the challenge of low SNR in EEG signals, we used a periodic flickering visual stimulus to elicit high-SNR responses, known as steady-state visual evoked potentials (SSVEPs). SSVEP responses are less susceptible to artifacts and exhibit high SNR (Norcia *et al* 2015), and hence, are widely used in human EEG, including the investigation of higher cognitive functions through frequency tagging (Vialatte *et al* 2010, Zhu *et al* 2010). It is important to note that SSVEP responses depend on the stimulus frequency (Alonso-Prieto *et al* 2013), with frequencies within alpha and beta range (10–25 Hz) eliciting the strongest responses (Ladouce *et al* 2022).

Second, leveraging the high temporal resolution of EEG, we developed a comprehensive predictive model to estimate V1 spiking activity using non-invasive EEG. Specifically, we used amplitude, phase, and phase-amplitude coupling (PAC) features of EEG bands to linearly model V1 spiking activity at each time point. Given that neural activity patterns may vary across cortical layers (Xing *et al* 2012, Mendoza-Halliday *et al* 2024), we also evaluated the model’s performance according to different cortical layers.

Our prediction model significantly exceeded chance performance, with markedly higher accuracy during SSVEP stimulus trials compared to non-SSVEP trials, and better prediction of activity in superficial cortical layers than deeper layers. Given the non-invasive nature of EEG in our study, this approach has the potential for direct translation to human EEG applications.

## 2. Methods

### 2.1. Subject, recording procedures, and apparatus

The subject was an adult male rhesus macaque (Macaca mulatta) weighing approximately 18 kg at the time of neural and behavioral data recordings. All experimental procedures were performed following the NIH Guide for the Care and Use of Laboratory Animals and the ARVO Statement for the Use of Animals in Ophthalmic and Vision Research, with approval from the Rutgers University Animal Care and Use Committee (Protocol ID: 201800245). Surgical implantation was conducted under fully aseptic conditions while the animal was under ketamine-induced isoflurane anesthesia. Ibuprofen and morphine analgesia were given as required post-surgery.

**Figure 1.**
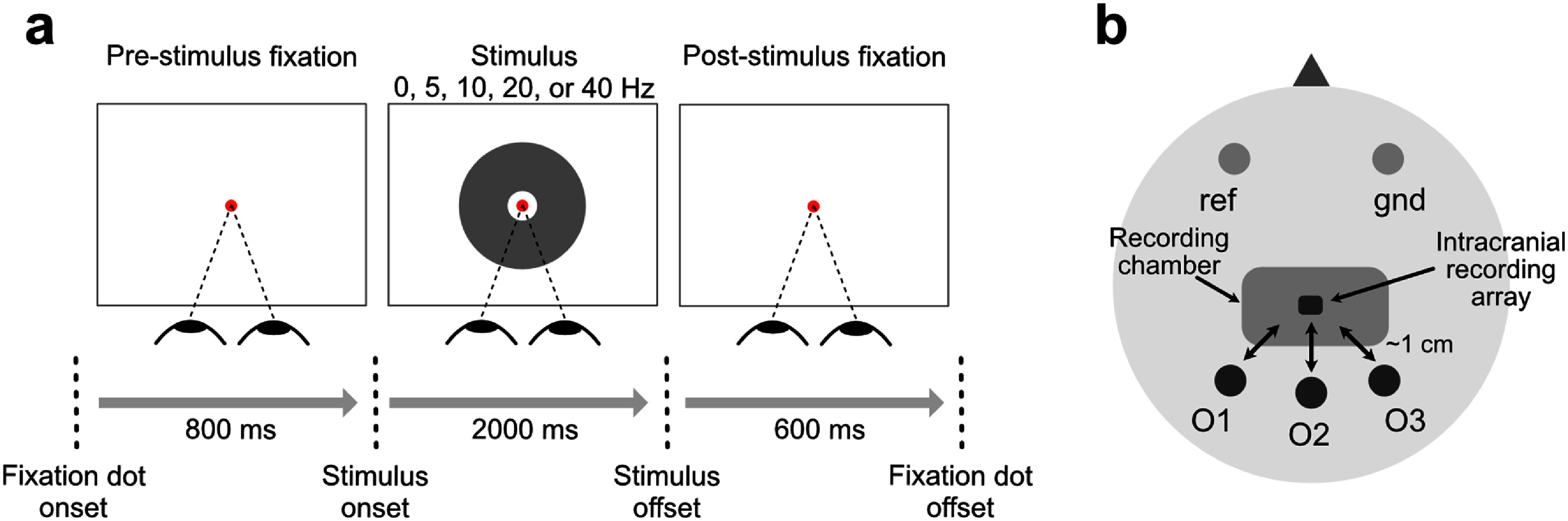
Behavioral task and EEG montage. (a) Schematic of behavioral task sequence, in which monkeys fixated on a central dot (not shown to scale) throughout three phases: pre-stimulus (800 ms), stimulus (2000 ms), and post-stimulus (600 ms), to receive a juice reward. During the stimulus phase, the screen flickers at one of the five frequencies (0, 5, 10, 20, or 40 Hz) to elicit steady-state visual evoked potentials (SSVEPs). (b) Schematic of the EEG electrode montage. Each dark-filled circle indicates a recording electrode on the occipital cortex (labeled O1, O2, O3), with the reference (ref) and ground (gnd) electrodes placed over the frontal cortex and indicated by a light gray circle. The round-edged rectangle indicates the position of the surgically implanted chamber used to protect the connectors of the intracranial recording array. The bidirectional arrows indicate the approximate distance (1 cm) between intracranial and EEG electrodes.

Neurophysiological signals were recorded while the animal engaged in a fixation task. Intracranial spiking activity was recorded using a 32-channel floating microarray, permanently implanted in the parafoveal region of the left hemisphere V1. The individual electrodes on the microarray (1.2 × 3.4 mm^2^) were spaced at 400 *μ*m both horizontally and vertically. Electrodes were distributed across four depths, with eight electrodes at each depth; the electrode length ranged from 0.6 to 1.5 mm relative to the dura surface, and the array was inserted perpendicular to the dura surface. EEG signals were concurrently recorded using three non-invasive scalp Ag/AgCl electrodes placed above the occipital lobe (see figure 1(b)), with online reference and ground EEG electrodes placed above the left and right frontal lobes, respectively.

During the recording sessions, the animal was seated comfortably in a standard primate chair without head restraint, approximately 57 cm away from a 120 Hz cathode-ray tube monitor. Eye movements were continuously monitored using an infrared video eye tracking system (EyeLink 1000; SR Research, Ottawa, Ontario, Canada) at a sampling rate of 1000 Hz. Stimulus presentation and eye tracking were controlled via our custom software Neurostim (Krekelberg *et al* 2022) and the Psychophysics Toolbox (Brainard 1997, Pelli 1997) in MATLAB (MathWorks Inc., Natick, MA). Neurophysiological, eye movement, and behavioral data were synchronously acquired with the Ripple system. Data analysis was performed using custom MATLAB scripts alongside standard analysis tools.

### 2.2. Behavioral task

The animal performed a dot-fixation task (figure 1(a)), in which it was required to maintain its gaze within 1.5° of a central fixation dot for the entire trial duration (3400 ms). A trial was considered successful when the animal fixated throughout three consecutive phases of the trial: pre-stimulus (800 ms), stimulus (2000 ms), and post-stimulus (600 ms).

During the pre-stimulus phase, the monkey initiated a trial by fixating on the central dot against a gray background for 800 ms, triggering the onset of the stimulus phase. In the stimulus phase, a donut-shaped stimulus (radius = 8.9 cm) centered around the fixation dot was displayed over the background. The donut flickered at one of five pseudorandomly selected temporal frequencies: 0, 5, 10, 20, or 40 Hz, referred to as trial conditions. In the 0 Hz condition, the donut appeared and remained stationary on the screen throughout the stimulus period, serving as an open-eye spontaneous condition without flicker. The flickering stimuli (5, 10, 20, and 40 Hz) were used to evoke SSVEPs, a response known to produce high SNRs in both EEG and intracranial signals (Norcia *et al* 2015).

During the post-stimulus phase, the background reverted to gray. A liquid reward was delivered contingent upon the animal maintaining fixation throughout all three phases. A 250 ms intertrial interval, during which no visual stimuli were present, followed each trial. Trials in which fixation was broken were aborted, and data from these trials were excluded from subsequent analyses.

### 2.3. Neurophysiological recordings and signals

Neural signals from the primary visual cortex (V1) and non-invasive EEG were recorded simultaneously. The primary goal of our acquisition setup was to record both intracranial (from V1) and extracranial (EEG) signals concurrently, while minimizing skull alteration to facilitate human-like, non-invasive EEG recordings. Therefore, we trained the animal to perform the task without a head-restraining device and kept the skull intact except at the site of chronic intracranial microarray implantation.

The intracranial electrode array implanted in V1 recorded the unfiltered wideband neural signals at a sampling rate of 30 kHz. We extracted the multi-unit spiking activity envelope (MUAe) from wideband signals using a procedure developed by (Supèr and Roelfsema 2005). This procedure involves the following steps sequentially: (1) band-pass filtering the wideband signal (500–5000 Hz), (2) rectifying the filtered signal, (3) applying the Hilbert transform to the rectified signal, and (4) downsampling to 1 kHz.

EEG was recorded at a sampling rate of 1 kHz with an online low-pass IIR filter of 500 Hz. Since the animal was not head-restrained, conductive adhesive gel (Ten20; Weaver and Company, Aurora, Colorado, USA) was applied to ensure stable electrode contact throughout the recording sessions. Before each session, the animal’s scalp was shaved and cleaned with saline and alcohol to achieve low impedance. Electrodes with impedance below 20 kOhm at the beginning of each recording session were included in subsequent analysis.

We applied bidirectional FIR digital filters of order 1000 on the recorded EEG signal to extract full-band and band-limited signals. We first removed power line noise using bidirectional bandpass FIR filters (60, 120, and 180 Hz). For full-band EEG, we then applied high-pass (1 Hz) and low-pass (250 Hz) filters. For band-limited signals we applied bandpass filters according to frequency band of interest: delta (2–4 Hz), theta (4–8 Hz), alpha (8–15), beta (15–30 Hz), low-gamma (30–60 Hz), gamma (60–120 Hz), and high-gamma (120–200 Hz). The full-band and band-limited signals were segmented into trials and aligned to the stimulus onset.

### 2.4. Electrode and trial selection

To identify functional MUAe electrodes from our chronic implant, we visually inspected the power spectrum of each electrode across multiple example sessions, focusing on spectral peaks at stimulus frequencies. Power spectra were computed over the 500–1500 ms window after stimulus onset using the fast Fourier transform. All cortical electrodes displayed prominent peaks at the stimulus frequencies. Hence, we included all 32 cortical electrodes in our analysis.

Next, for each session, we assessed whether the signal measured on EEG electrode remained responsive to task stimuli. This step was important because the animal was not head-restricted, which sometimes led to excessive head movement. Such movement could cause EEG electrodes to shift or loosen, resulting in poor contact and compromised signal quality within the session. To quantify the signal quality of each EEG electrode in each session, we calculated the SNR, defined as the ratio of evoked power at the stimulus frequency to the average power at the two neighboring frequencies. Only EEG electrodes with an SNR > 1.5 for at least one stimulus condition in a session were retained for further analysis. Applying these criteria resulted in 31 EEG electrodes across 14 sessions, yielding a total of 992 EEG-MUAe electrode pairs used in our analysis.

Artifact trials were identified using both EEG and LFP signals. Based on LFP, trials were marked as artifacts if they met any of the following criteria: (1) high synchronization relative to other trials (*z* > 4) across more than 25% of electrodes; (2) outlier signals relative to other trials (*z* > 4) in at least two electrodes; or (3) LFP magnitude exceeding 500 *μ*V in more than two electrodes. Based on EEG, artifact trials were marked based on two criteria: (1) outlier signal relative to other trials (*z* > 4) at any EEG electrode; or (2) EEG magnitude exceeding 100 *μ*V for over 10 ms in any electrode. Trials marked by either method were excluded from further analysis. This resulted in 4318 trials used for further analysis, averaging 308 trials per session (SD = 105).

### 2.5. Data analysis

We focused our analysis on identifying relationships between MUAe and EEG, aiming to develop a comprehensive linear model to predict V1 spike dynamics from EEG. For all our analyses below, we used a 400–1800 ms timeseries signal following stimulus onset, either the single-trial timeseries (‘single-trial’) or the trial-averaged timeseries (‘trial-averaged’). All analyses were performed in MATLAB (R2019b).

#### 2.5.1. Correlation across time

To quantify the relationship between EEG and MUAe signals, we calculated Pearson’s correlation coefficients between their time series, separately for each stimulus condition. These correlations were computed for both full-band and band-limited EEG across all EEG-MUAe electrode pairs.

#### 2.5.2. PAC

We used the Modulation Index (MI, Tort *et al* 2008) to quantify the coupling between the phase of EEG signals and the amplitude of both EEG and MUAe. To calculate MI, phase values were binned into 18 intervals of 20° from −180° to 180°. The corresponding EEG amplitude or MUAe was averaged for each phase bin to construct the phase-amplitude distribution. Following the methodology of Hülsemann *et al* (2019), we then computed the MI as the Kullback–Leibler divergence between the observed phase-amplitude distribution and a uniform distribution. We chose MI over alternatives such as phase-locking value or mean vector length because MI can detect biphasic coupling and is less susceptible to confounding factors like SNR and data length (Hülsemann *et al* 2019).

#### 2.5.3. MUAe prediction model

The flowchart in supplementary figure 1 illustrates the steps we used to construct a model that predicted the instantaneous MUAe at each time point from EEG spectrotemporal features. These features included the analytic amplitude, the instantaneous phase of seven frequency bands, and PAC between a subset of bands. Specifically, the fitted model was

\begin{align*} y\left( t \right) &amp;= {\beta _0} + \mathop \sum \limits_{k = 1}^7 {\beta _{A,k}}{A_k}\left( t \right) + {\mathop \sum \limits_{k = 1}^7} \left( {\beta _{s,k}}\sin {\phi _k}\left( t \right)\right.\nonumber\\ &amp;\left.\quad + {\beta _{c,k}}\cos {\phi _k}\left( t \right) \right) \nonumber\\ &amp;\quad+ {\mathop \sum \limits_{i = 4}^7} {\mathop \sum \limits_{j = 1}^3} \left( {\beta _{s,i,j}}{A_i}\left( t \right)\sin {\phi _j}\left( t \right)\right.\nonumber\\ &amp;\left.\quad + {\beta _{c,i,j}}{A_i}\left( t \right)\cos {\phi _j}\left( t \right) \right) + \varepsilon \left( t \right).\end{align*}

Here $y\left( t \right)$ is the instantaneous MUAe at time $t$. ${A_k}\left( t \right)$ and ${\phi _k}\left( t \right)$ are the analytic amplitude and instantaneous phase of EEG frequency band k (bands ordered low → high; *k* = 1 is delta, *k* = 2 is theta, *k* = 3 is alpha, *k* = 4 is beta, *k* = 5 is low-gamma, *k* = 6 is gamma, and *k* = 7 is high-gamma); $\sin {\phi _k}\left( t \right)$ and $\cos {\phi _k}\left( t \right)$ are the phase predictors used to linearize the circular phase variable. The first sum captures linear contributions of band amplitudes, the second sum captures the linear contribution of band phases, where each circular phase is transformed into sine and cosine components to enable linear modeling. The double sum includes amplitude-phase interaction terms between amplitude bands $i = 4 - 7$ (beta, low-gamma, gamma, and high-gamma) and phase bands $j = 1 - 3$ (delta, theta, alpha), selected based on PAC analysis (figure 5). ${\beta _0}$ is the intercept, terms are the model coefficients, and $\varepsilon \left( t \right)$ denotes residual error. The phase-related beta coefficients are reported as the Euclidean norm of their sine and cosine coefficient pairs (e.g. $\left| {{\beta _{phase,k}}} \right| = \sqrt {\beta _{s,k}^2 + \beta _{c,k}^2} $). All predictors were *z*-scored across samples prior to model fitting.

We extended the model to include the stimulus phase as an additional predictor. The screen flickered sinusoidally at one of five temporal frequencies (0, 5, 10, 20, 40 Hz; 0 Hz = non-flicker). Motivated by the hypothesis that repetitive stimulation may enhance EEG-to-spike predictability (Mazzoni *et al* 2010), we evaluated whether the phase of the stimulus improves model predictions. The extended model is as follows:

\begin{align*} y\left( t \right) &amp;= \left( {{\text{Eq}}.1} \right) + \left( {\beta _{s,{\text{stim}}}}\sin {\phi _{{\text{stim}}}}\left( t \right)\right.\nonumber\\ &amp;\left.\quad+ \text{ }{\beta _{c,{\text{stim}}}}\cos {\phi _{{\text{stim}}}}\left( t \right) \right).\end{align*}

Relative to the original model (equation (1)), this extended model is identical except for the addition of the stimulus-phase predictors $\sin {\phi _{{\text{stim}}}}\left( t \right)$ and $\cos {\phi _{{\text{stim}}}}\left( t \right)$, indicating the linearized encoding of the circular phase variable, ${\phi _{{\text{stim}}}}$.

Model performance was evaluated using a 5-fold cross-validation procedure. In each iteration, time samples from 4 folds were used to train the model with LASSO regression, and the trained coefficients were applied to predict MUAe in the held-out test samples. The average model performance across all five folds is reported.

To determine the optimal regularization parameter (*λ*) for the LASSO regression, we performed a nested 5-fold cross-validation on the training data, following L1 norm criterion (Hastie *et al* 2015). During this process, a range of *λ* values was evaluated, and the *λ* that minimized the average mean squared error across the inner folds was selected as the optimal regularization parameter. The coefficients corresponding to this *λ* were then estimated using the entire training set and used to predict MUAe in the test data during each fold. This approach ensured an unbiased and robust estimate of model accuracy on unseen data.

#### 2.5.4. Statistical analysis

Since we recorded from multiple electrodes simultaneously—32 MUAe electrodes and 3 EEG electrodes—the measurements are likely not fully independent. Measurements from electrodes within the same session may be partially correlated, which means the usual assumptions of independence in many statistical tests do not strictly hold in this dataset. To address this, we used linear mixed-effect models, with random intercept effects for each EEG-MUAe electrode pair to account for potential dependencies between electrode pairs and a separate random intercept effect for each session to account for repeated measurements within a session.

Specifically, we used MATLAB’s *fitlme.m* function to implement mixed-effect models, which allowed us to evaluate the statistical measures of EEG–MUAe correlation coefficients and model performance metrics. To account for the effect of repeated measurements on the degrees of freedom and corresponding p-values, we calculated the degrees of freedom using the Satterthwaite approximation method. Code for data analysis and the data used in this manuscript are available at https://osf.io/vq5th.

## 3. Results

The primary objective of this study was to predict V1 spike dynamics from non-invasive EEG recordings at millisecond resolution. We simultaneously recorded multi-unit spiking activity envelopes (MUAe) from V1 and scalp EEG over the occipital cortex in a macaque monkey (figure 1(b)). Neural data were collected during a fixation task in which the screen flickered at one of five temporal frequencies (0, 5, 10, 20, and 40 Hz) per trial (figure 1(a)), with the frequency selected pseudorandomly on each trial. These stimuli evoked high SNR steady-state visual potentials (SSVEPs; Norcia *et al* 2015). For all analyses, we used signals from 400 to 1800 ms post-stimulus onset to minimize transient effects related to stimulus onset and offset.

### 3.1. Correlations between MUAe and full-band EEG are weak and stimulus-dependent

**Figure 2.**
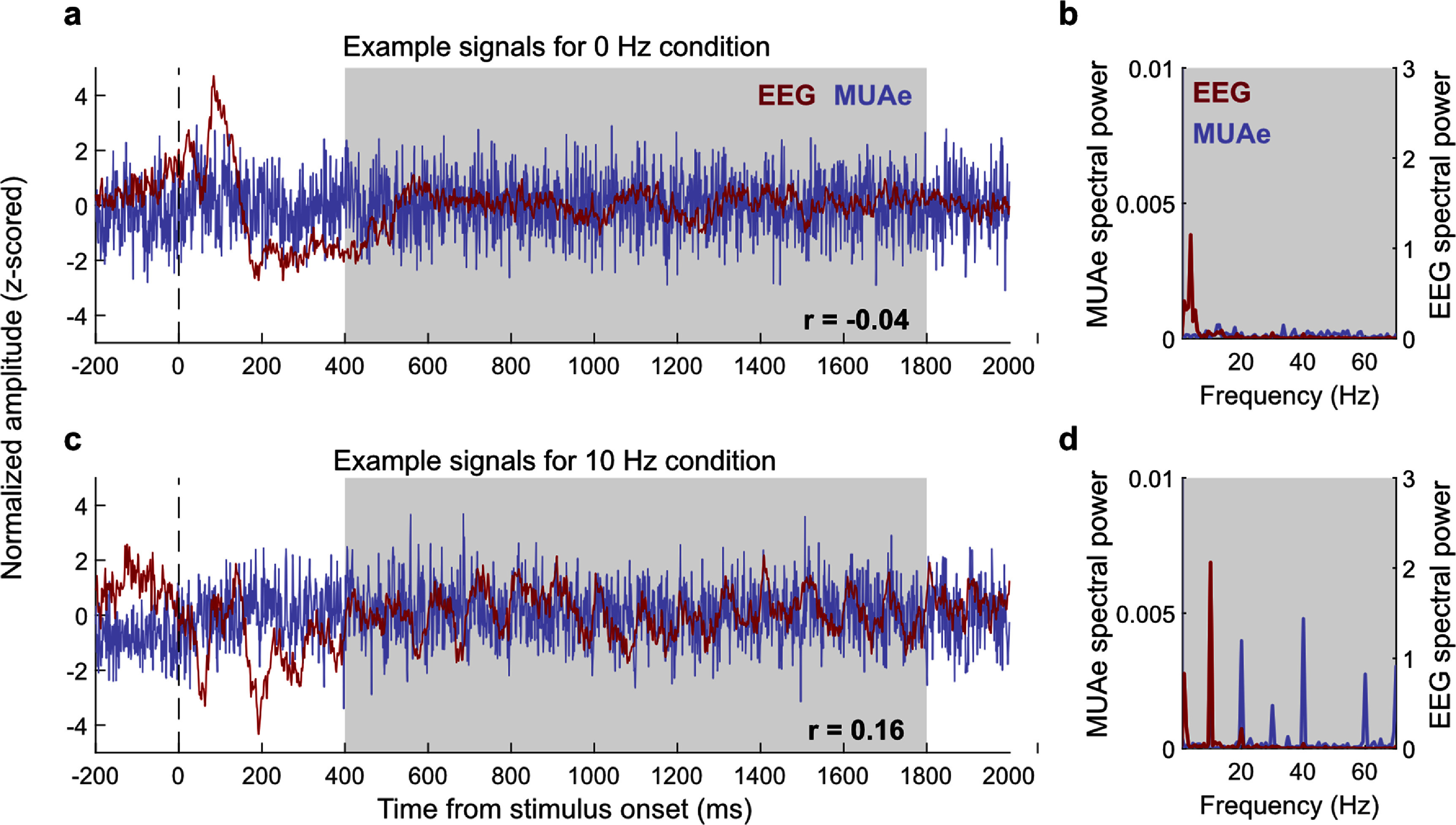
Time-series and spectral representations of neural signals from representative electrodes. (a) Neural signals during the spontaneous (0 Hz) condition. Blue and red traces indicate time-series averaged across trials for MUAe and EEG signal, respectively; correlation between these signals is *r* = −0,04. A clear onset response can also be seen in the EEG signal. (b) Power spectra of the signals in panel (a) computed over the 400–1800 ms time window following stimulus onset (shaded gray region). (c) Same as (a), but for trials with a 10 Hz flickering stimulus. (d) Same as (b), but for the 10 Hz stimulus condition.

**Figure 3.**
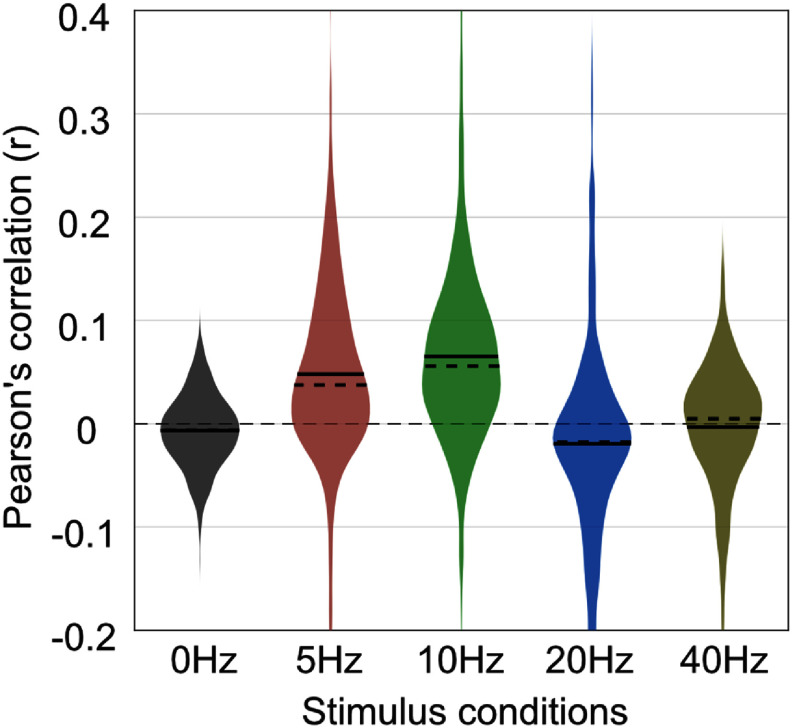
Correlation coefficients between trial-averaged EEG and MUAe signals. Each violin plot displays the distribution of correlation values across all EEG–MUAe electrode pairs and sessions. The results indicate that the correlation is non-significant for the spontaneous (0 Hz) condition (*p* = 0.35), but significant for flickering stimuli (5 Hz, *p* = 1 × 10^−4^; 10 Hz, *p* = 3 × 10^−6^; 20 Hz, *p*= 0.03), except for 40 Hz (*p* = 0.57). This suggests that the relationship between EEG and MUAe depends on stimulus frequency.

To evaluate a direct relationship between EEG and MUAe we first calculated Pearson’s correlation between full-band EEG and MUAe timeseries signals averaged across trials (i.e. trial-averaged timeseries). Figure 2 shows example EEG and MUAe timeseries signals and their corresponding spectrum. In this example, the correlation coefficient for the spontaneous (0 Hz) and flickering (10 Hz) conditions is −0.004 and 0.16, respectively, indicating a stronger correlation between the EEG and MUAe induced by flickering stimuli. Figure 3 shows the distribution of correlation coefficients across all EEG-MUAe pairs for each stimulus condition. Similar to the pattern seen in example signals, correlations for the 0 Hz condition was small and non-significant (*r* = −0.008 ± 0.010, *p* = 0.37) while the10 Hz condition showed significant positive correlations (*r* = 0.06 ± 0.01, *p* = 3.0 × 10^−6^). The correlation was positive for 5 Hz (*r* = 0.05 ± 0.01, *p* = 1.0 × 10^−4^), slightly negative for 20 Hz (*r* = −0.02 ± 0.01, *p* = 0.03) and non-significant for 40 Hz (*r* = −0.005 ± 0.010, *p* = 0.57). These results indicate that the strength of the EEG–MUAe relationship depends strongly upon the stimulus frequency, corroborating earlier findings (Snyder and Smith 2015).

### 3.2. Relationship between EEG and V1 spiking depends on frequency bands in EEG

**Figure 4.**
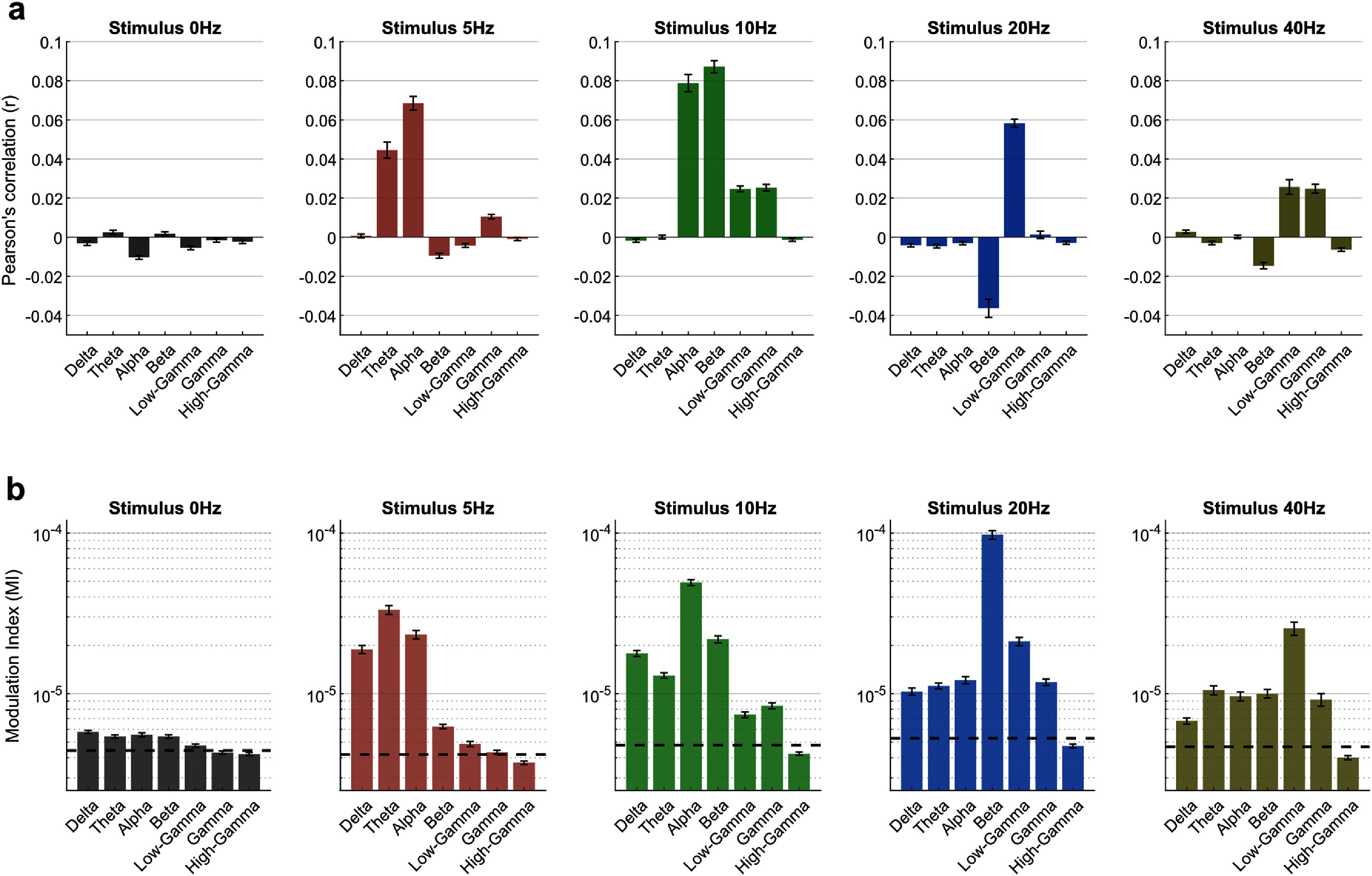
Relationship between MUAe and EEG band-limited signals. (a) Pearson’s correlation coefficients calculated across time between trial-averaged MUAe and EEG signals filtered into canonical frequency bands: delta (2–4 Hz), theta (4–8 Hz), alpha (8–15 Hz), beta (15–30 Hz), low-gamma (30–60 Hz), gamma (60–120 Hz), and high-gamma (120–200 Hz). Each bar represents the mean correlation across all electrode pairs recorded over sessions, for each stimulus condition (0, 5, 10, 20, and 40 Hz flicker frequency). The relation between MUAe and EEG bands amplitude varied across frequencies and stimulus conditions. (b) Modulation Index (MI; see Methods) quantifies phase-amplitude coupling (PAC) between the amplitude of MUAe and the phase of the EEG signal. Each bar displays the mean MI over all electrode pairs, for each stimulus condition, with dashed lines indicating the mean MI obtained from shuffled phase data (iterated 1000 times). Error bars represent the standard error of the mean (SEM).

We then investigated how the phase and amplitude of EEG bands—delta (2–4 Hz), theta (4–8 Hz), alpha (8–15 Hz), beta (15–30 Hz), low-gamma (30–60 Hz), gamma (60–120 Hz), and high-gamma (120–200 Hz)—relate to V1 MUAe. Specifically, we computed time series of the band-limited amplitude and phase and determined their correlation with the MUAe time series (figure 4(a)).

In the non-flicker spontaneous (0 Hz) condition, correlations across all bands were weak and near zero, with slight negative trends observed in delta (*r* = −0.003 ± 0.002, *p* = 0.046), alpha (*r* = −0.01 ± 0.002, *p* = 7.1 × 10^−8^), and low-gamma (*r* = −0.005 ± 0.002, *p* = 9.4 × 10^−4^). During flickering stimuli at 5, 10, 20, and 40 Hz, the significant correlations tended to occur in bands resonant with the stimulus frequency or its harmonics.

For the 5 Hz stimulus, positive correlations were observed in the theta (*r* = 0.043 ± 0.005, *p* = 1.5 × 10^−7^) and alpha (*r* = 0.067 ± 0.005, *p* = 8.8 × 10^−11^) along with some negative trend in the beta band (*r* = −0.012 ± 0.005, *p* = 0.037). The 10 Hz condition showed higher positive correlations, particularly in the alpha (*r* = 0.083 ± 0.007, *p* = 3.3 × 10^−10^), beta (*r* = 0.091 ± 0.007, *p* = 6.9 × 10^−11^), low-gamma (*r* = 0.027 ± 0.007, *p* = 6.3 × 10^−4^) and gamma (*r* = 0.028 ± 0.007, *p* = 5.0 × 10^−4^) bands. Interestingly, in the 20 Hz condition, two neighboring bands showed significant correlations with opposite trends: negative in the beta band (*r* = −0.039 ± 0.005, *p* = 3.3 × 10^−7^), positive in the low-gamma band (*r* = 0.056 ± 0.005, *p* = 6.2 × 10^−10^). Finally, the 40 Hz stimulus elicited positive correlations in the low-gamma (*r* = 0.021 ± 0.006, *p* = 0.005) and gamma (*r* = 0.020 ± 0.006, *p* = 0.008) bands, but negative correlations in the beta band (*r* = −0.020 ± 0.006, *p* = 0.008). Compared to these trial-averaged signals correlations, EEG-MUAe correlations were weaker at the single-trial level but retained stimulus- and band-dependent patterns (supplementary figure 2). Overall, these results indicated that, depending on the stimulus, all EEG frequency bands potentially contain information on the MUAe.

We next performed a similar analysis to assess whether EEG phase is related to MUAe activity. Specifically, we quantified PAC between the EEG phase and MUAe using the Modulation Index (MI; Tort *et al* 2008). Similar to the correlation results above, PAC between EEG bands and MUAe varied across stimulus conditions. For the spontaneous (0 Hz) condition, coupling was generally weak, with values only slightly above the shuffled values for the delta to beta bands. In contrast, flickering stimuli showed higher MI for certain bands relative to shuffled data (figure 4(b)). Specifically, the 5 Hz stimulus showed increased MI for delta to beta bands, while the 10, 20, and 40 Hz stimuli showed high MI for all bands except high-gamma.

These results highlight the frequency-specific nature of the relationship between EEG and MUAe, and emphasize the importance of examining both phase and amplitude of each EEG band when studying EEG-spike relationships, extending previous findings (Whittingstall and Logothetis 2009).

### 3.3. Amplitude and phase of EEG bands predict V1 spike dynamics

Leveraging observations from the correlation and PAC analysis, we implemented a cross-validated, L1-regularized, regression model to estimate MUAe at each time point from EEG features, including amplitudes, phases, and interaction terms of EEG bands (equation (1)). These interaction terms accounted for PAC between EEG amplitude and phase, a phenomenon observed in several human studies (Canolty and Knight 2010). We hypothesized that PAC between EEG bands might provide unique information beyond what is available from amplitude or phase alone, thereby potentially enhancing MUAe prediction accuracy (Canolty and Knight 2010, Fell and Axmacher 2011).

**Figure 5.**
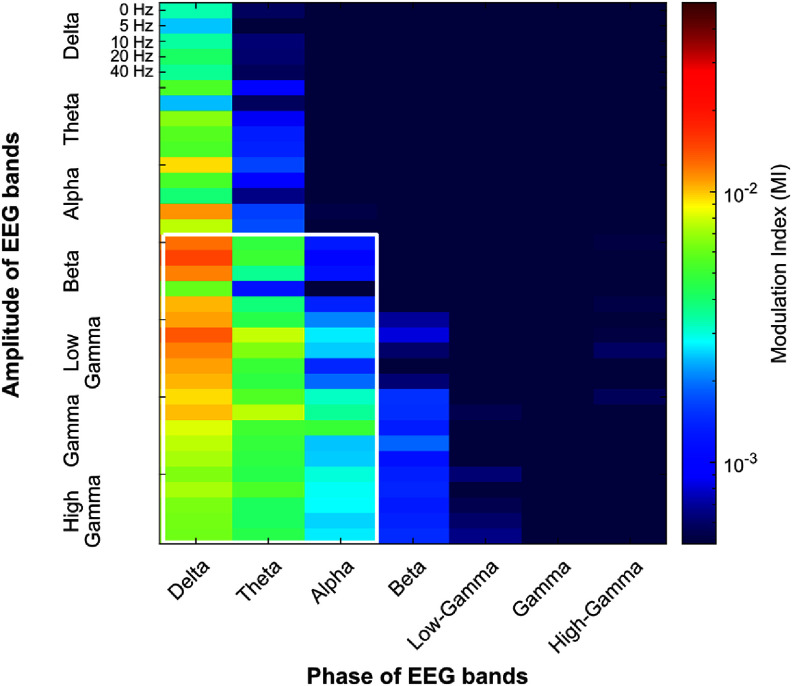
Phase-amplitude coupling (PAC) between EEG bands. The color axis indicates the PAC, measured by the Modulation Index (MI), between phases (*x*-axis) and amplitudes of EEG bands (*y*-axis) for each stimulus condition (0, 5, 10, 20, and 40 Hz). The MI values indicate the strength of phase-dependent amplitude modulation, with higher values reflecting stronger PAC. The white rectangle highlights the specific phase-amplitude pairs used as interaction terms in the model for predicting MUAe (see equation (1). Based on these observations, 12 amplitude-phase interaction terms—pairings of three lower-frequency phase bands with four higher-frequency amplitude bands—were included in the prediction model.

We first assessed which pairs showed strong PAC by calculating MI for each phase-amplitude pair. As shown in figure 5 (white box), the phase of low-frequency bands (delta, theta, and alpha) was coupled with the amplitude of high-frequency bands (beta, low-gamma, gamma, and high-gamma). Based on this observation, we included 12 interaction terms in our model: pairing the phase of three bands (delta, theta, and alpha) with the amplitude of four bands (beta, low-gamma, gamma, and high-gamma) (equation (1)).

The model was 5-fold cross-validated across time samples. Pearson’s correlations (r) between the estimated MUAe and original MUAe for the left-out samples quantified model performance, and we report the average correlation across folds.

**Figure 6.**
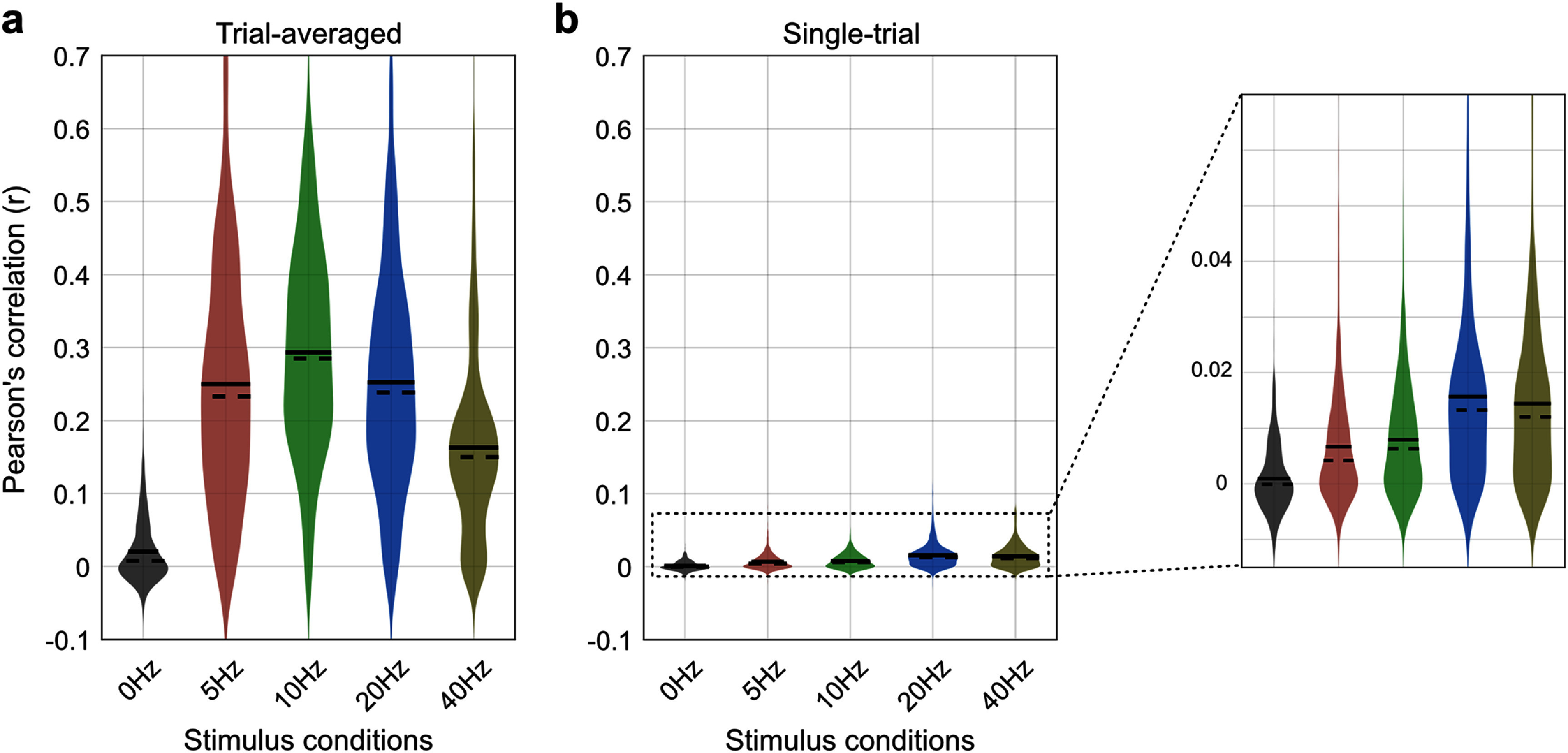
Model prediction accuracy quantified by Pearson’s correlation between the time series of original and predicted MUAe. (a) Violin plots representing the distribution of correlation coefficients across all EEG–MUAe electrode pairs and sessions for trial-averaged MUAe signals. The model performed significantly above chance for flickering stimuli (5 Hz, *r* = 0.26, *p* = 5 × 10^−10^; 10 Hz, *r* = 0.30, *p* = 4 × 10^−11^; 20 Hz, *r* = 0.26; *p* = 5 × 10^−11^; 40 Hz, *r* = 0.17; *p* = 5 × 10^−7^); the correlation for the non-flickering (0 Hz) condition was not significant (*p* = 0.35). (b) Same as (a), but using single-trial MUAe signals. The inset shows an enlarged *y*-axis for clearer visualization. The correlations were generally lower but still significant for flickering stimuli(5 Hz, *r* = 0.006, *p* = 2 × 10^−5^; 10 Hz, *r*= 0.008, *p* = 2 × 10^−6^; 20 Hz, *r* = 0.016, *p* = 5 × 10^−12^; 40 Hz, *r* = 0.014, *p* = 3 × 10^−11^); the correlation for 0 Hz stimuli remained non-significant (*p* = 0.55).

Figure 6(a) shows the distribution of correlations across EEG-MUAe electrode pairs and sessions for trial-averaged signals. The model performed well for all flickering stimuli (5, 10, 20, and 40 Hz) but not for non-flickering 0 Hz stimuli (*r* = 0.02 ± 0.022, *p* = 0.35). The best performance was observed for the 10 Hz stimulus (*r* = 0.30 ± 0.022, *p* = 4.0 × 10^−11^), followed by 20 Hz (*r* = 0.26 ± 0.022, *p* = 4.8 × 10^−10^), 5 Hz (*r* = 0.26 ± 0.022, *p* = 5.1 × 10^−10^), and 40 Hz (*r* = 0.17 ± 0.022, *p* = 5.1 × 10^−7^).

Compared to trial-averaged signals, model performance was worse for trial-level signals (figure 6(b)). At the trial-level, the model performed worst for 0 Hz stimulus (*r* = 0.001 ± 0.001, *p* = 0.55) but better for flickering stimuli (5 Hz, *r* = 0.006 ± 0.001, *p* = 2.0 × 10^−5^; 10 Hz, *r* = 0.008 ± 0.001, *p* = 1.6 × 10^−6^; 20 Hz, *r* = 0.016 ± 0.001, *p* = 4.9 × 10^−12^; 40 Hz, *r* = 0.014 ± 0.001, *p* = 2.8 × 10^−11^). A closer look at the correlations revealed that the 20 and 40 Hz stimuli elicited higher correlations than 5 and 10 Hz stimuli (see inset in figure 6(b)).

To compare the performance of our model relative to direct EEG–MUAe correlations (figure 3), we calculated the difference between the correlation coefficient of the EEG–MUAe pair and that of the estimated versus actual MUAe. Our model showed better correlations than direct EEG–MUAe in all stimulus conditions, for both trial-averaged (0 Hz, Δ*r* = 0.027, *p* = 8.3 × 10^−6^; 5 Hz, Δ*r* = 0.213, *p* < 1.0 × 10^−30^; 10 Hz, Δ*r* = 0.239, *p* < 1.0 × 10^−30^; 20 Hz, Δ*r* = 0.278, *p* < 1.0 × 10^−30^; 40 Hz, Δ*r* = 0.170, *p* < 1.0 × 10^−30^) and single-trial signals (0 Hz, Δ*r* = 0.001, *p* = 0.065; 5 Hz, Δ*r* = 0.005, *p* < 2.7 × 10^−14^; 10 Hz, Δ*r* = 0.005, *p* < 9.9 × 10^−16^; 20 Hz, Δ*r* = 0.016, *p* < 1.0 × 10^−30^; 40 Hz, Δ*r* = 0.012, *p* < 1.0 × 10^−30^).

**Figure 7.**
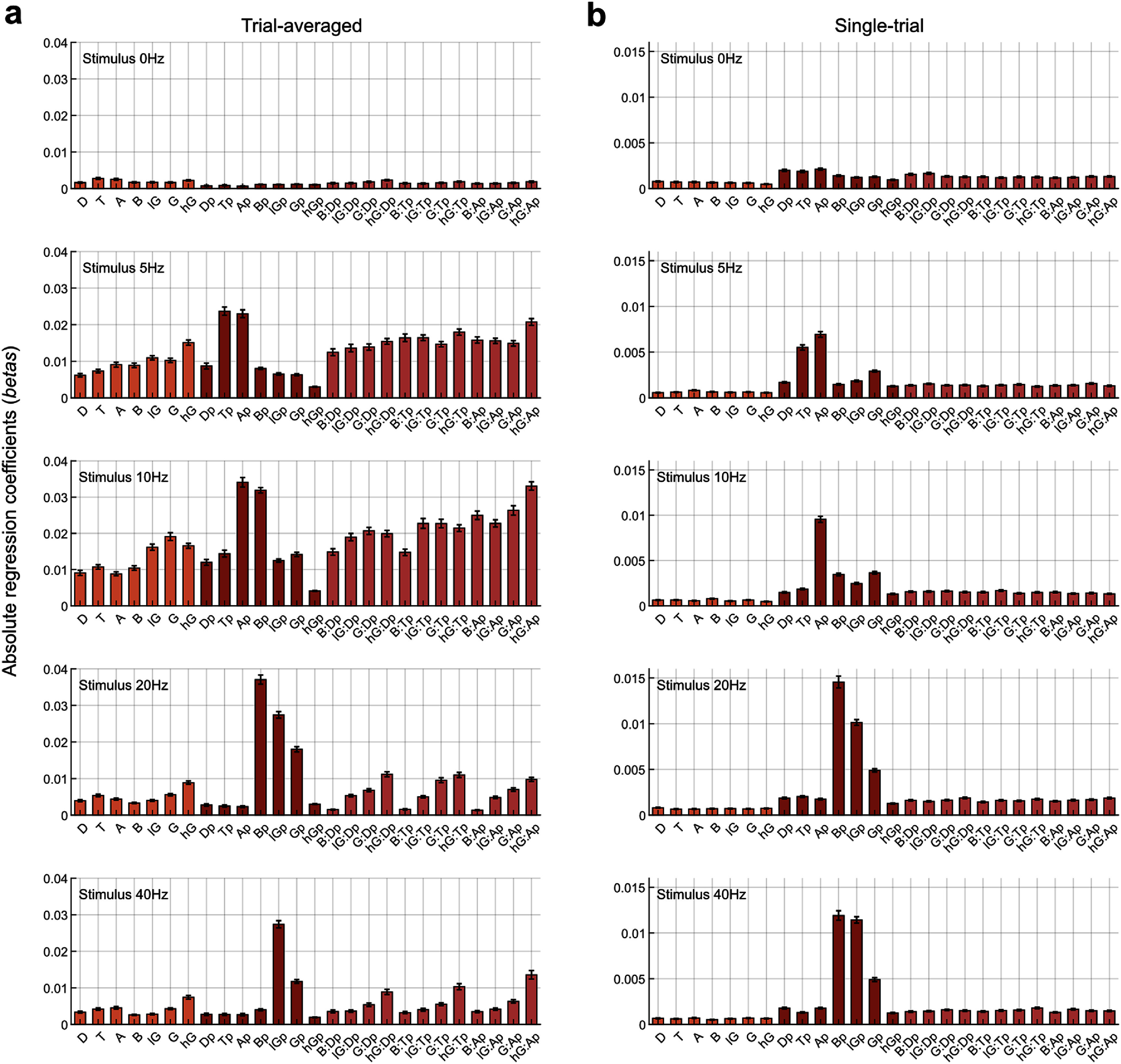
Regression coefficients of variables in the predictive model (equation (1)). (a) Mean regression coefficients across all EEG–MUAe electrode pairs for trial-averaged signals, shown separately for each stimulus condition (0, 5, 10, 20, and 40 Hz). (b) Same as (a), but for single-trial signals. Error bars indicate the standard error of the mean (SEM). Variable notation: Amplitude variables are indicated by a capital letter representing the frequency band: D (delta, 2–4 Hz), T (theta, 4–8 Hz), A (alpha, 8–15 Hz), B (beta, 15–30 Hz), lG (low-gamma, 30–60 Hz), G (gamma, 60–120 Hz), hG (high-gamma, 120–200 Hz). Phase variables are indicated by the same capital letter with a suffix ‘p’ (e.g. Dp for delta phase). Interaction terms between an amplitude and a phase variable are indicated by a colon (:) (e.g. B:Dp for interaction between beta amplitude and delta phase). These coefficients reflect the relative contributions of each feature to predicting MUAe from EEG signals across different stimulus conditions.

To better understand the relative contribution of amplitude, phase, and interaction terms used in the model, we compared the absolute values of the regression coefficients (β; see Methods) for these (*z*-scored) predictor variables. We grouped absolute betas of all predictor variables in three categories: amplitude of seven bands, phase of seven bands, and coupling indicated by interaction terms of the model (figure 7), and compared them using a linear mixed-effect model (see Methods).

Comparing amplitude and phase categories for trial-averaged signals revealed significant differences between them: whereas the spontaneous 0 Hz condition showed greater contribution of amplitude variables (difference in absolute beta coefficient, Δ*b* = 0.001, *p* = 1.0 × 10^−12^), flickering conditions showed greater contribution of phase variables (5 Hz, Δ*b* = − 0.002, *p* = 1.6 × 10^−4^; 10 Hz, Δ*b* = − 0.005, *p* = 7.4 × 10^−9^; 20 Hz, Δ*b* = −0.009, *p* = 3.6 × 10^−20^; 40 Hz, Δ*b* = − 0.003, *p* = 1.8 × 10^−10^). Similarly, comparison of amplitude and coupling categories showed greater betas for amplitude for 0 Hz condition (Δ*b* = 0.0004, *p* = 1.4 × 10^−7^), but the reverse for flickering conditions (5 Hz, Δ*b* = −0.006, *p* = 1.5 × 10^−15^; 10 Hz, Δ*b* = −0.009, *p* = 4.8 × 10^−14^; 20 Hz, Δ*b* = −0.001, *p* = 7.8 × 10^−5^; 40 Hz, Δ*b* = −0.002, *p* = 2.5 × 10^−7^). Overall, these results suggest that, for trial-averaged EEG-to-MUAe estimations, amplitude variables contributed more in spontaneous conditions, whereas phase and coupling variables contributed more in flickering conditions.

Interestingly, for single-trial estimations, phase variables always contributed more than amplitude variables for all stimulus conditions (0 Hz, Δ*b* = −0.001, *p* = 1.2 × 10^−35^; 5 Hz, Δ*b*= −0.003, *p* = 1.87 × 10^−27^; 10 Hz, Δ*b* = −0.003, *p* = 3.63 × 10^−30^; 20 Hz, Δ*b* = −0.005, *p* = 2.95 × 10^−28^; 40 Hz, Δ*b* = −0.004, *p* = 1.82 × 10^−23^). Similar was the case for the amplitude and coupling comparison (0 Hz, Δ*b* = −0.0007, *p* = 6.9 × 10^−31^; 5 Hz, Δ*b* = −0.0007, *p* = 6.7 × 10^−15^; 10 Hz, Δ*b*= −0.0009, *p* = 2.1 × 10^−17^; 20 Hz, Δ*b* = −0.0009, *p* = 8.0 × 10^−12^; 40 Hz, Δ*b* = −0.0009, *p* = 9.3 × 10^−11^). These results suggest that EEG phase and coupling are almost always more informative for MUAe than EEG amplitude.

### 3.4. Phase of stimulus frequency improves EEG-to-spike predictions

As mentioned above, Whittingstall and Logothetis (2009) found that the phase of EEG delta band (2–4 Hz) can help predict spiking activity in V1 during naturalistic movie viewing tasks. Mazzoni *et al* (2010) explained this by suggesting that V1 is adapted to naturalistic sensory stimuli, which tend to induce oscillations within the delta frequency range. They argued that this is why EEG delta phase provides valuable information about V1 spiking activity. Building on this idea, Mazzoni *et al* (2010) hypothesized that any stimulus or stimulation causing V1 activity to oscillate in a regular, periodic manner could improve the prediction of V1 spiking. Specifically, they proposed that the phase of stimulus frequencies could serve as a key predictor of V1 spiking activity.

**Figure 8.**
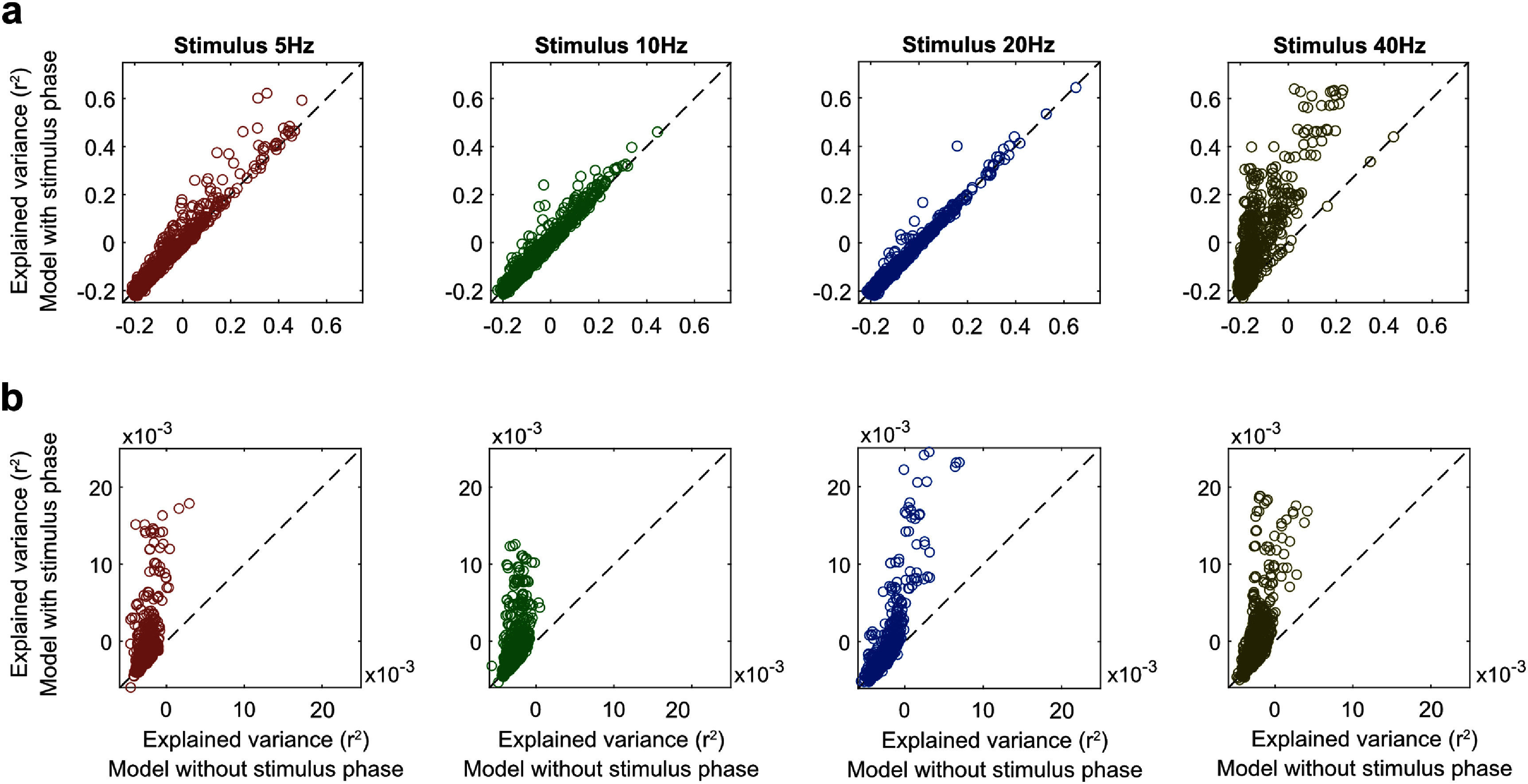
Effect of stimulus phase on EEG-to-MUAe prediction performance. Scatter plots comparing the explained variance (adjusted *r*^2^) of prediction models with and without the stimulus phase as a predictor (equation (1) vs. equation (2)). The *x*-axis shows the model’s performance when stimulus phase is excluded (equation (1)), and the *y*-axis shows performance when stimulus phase is included (equation (2)). Each point represents predictions for an EEG-MUAe electrode pair in a single session. (a) Predictions based on trial-averaged MUAe and EEG signals. (b) Predictions based on single-trial MUAe and EEG signals. The diagonal line indicates equal performance in both models. Points above the diagonal demonstrate improved prediction accuracy when stimulus phase is included in the model. Results show significant performance improvements across stimulus conditions at the single-trial level, supporting the hypothesis that stimulus phase enhances EEG-to-MUAe predictions.

To test this hypothesis, we included the stimulus phase in the prediction model (equation (2)) and compared the explained variance (adjusted *R*^2^) of this model with the explained variance of the model without the stimulus phase (equation (1)). We found that including stimulus phase significantly improved the model performance, particularly at a single-trial level (figure 8). For trial-averaged estimations, only 40 Hz condition showed significant improvement (difference in adjusted *r*^2^, Δ*r*^2^ = 0.087, *p* = 1.3 × 10^−10^); the model performance was not different for any other stimulus condition (5 Hz, Δ*r*^2^ = 0.002, *p* = 0.77; 10 Hz, Δ*r*^2^ = 0.003, *p* = 0.70; 20 Hz, Δ*r*^2^ = −0.007, *p* = 0.29). For trial-level estimations, models with stimulus phase performed better for all stimulus conditions (5 Hz, Δ*r*^2^ = 0.0012, *p* = 0.0018; 10 Hz, Δ*r*^2^ = 0.0015, *p* = 0.0003; 20 Hz, Δ*r*^2^ = 0.0011, *p* = 0.0044; 40 Hz, Δ*r*^2^ = 0.0026, *p* = 7.3 × 10^−8^), supporting the hypothesis proposed by Mazzoni *et al* (2010).

### 3.5. Spiking activity predictions are better at superficial than deep layers

Multiple studies have shown that spiking and LFP activity show distinct patterns across cortical layers (Mitzdorf 1985, Bastos *et al* 2018, Mendoza-Halliday *et al* 2024). This suggests that our EEG-to-MUAe model prediction performance may also vary across cortical layers in V1. To investigate this, we compared model prediction accuracy between MUAe recorded at different cortical depths.

We recorded MUAe from 32 electrodes placed at four equidistant depths, putatively spanning superficial (2/3) to deep cortical layers (4, 5, 6) in V1. To directly compare the model performance at superficial versus deep layers, we grouped the electrodes into two categories: shallow (covering layers 2/3) and deep (covering layers 4–6). The laminar placement of these electrodes was confirmed using the methodology developed by Mendoza-Halliday *et al* (2024).

**Figure 9.**
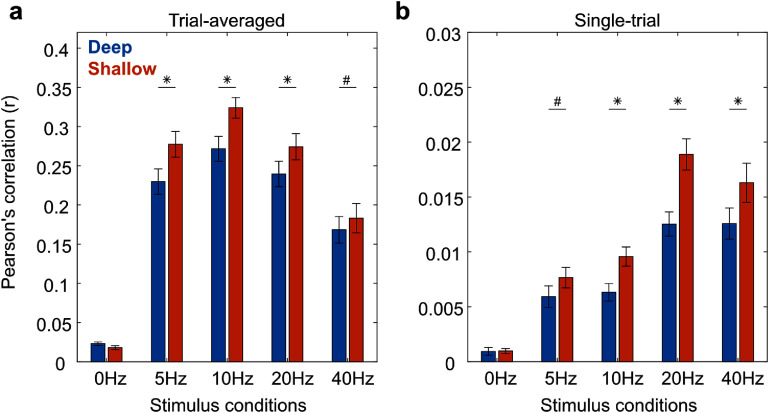
EEG-to-MUAe prediction accuracy for deep and superficial cortical layers in V1. Bar plots show the average model prediction accuracy (Pearson’s correlation), separately for electrodes targeting deep (blue bars) and shallow (orange bars) cortical layers. Significant differences between deep and superficial sites are indicated by * (*p* < 0.05) and # (*p* < 0.10). (a) Prediction performance based on trial-averaged EEG and MUAe signals. (b) Prediction performance based on single-trial EEG and MUAe signals. These comparisons indicate layer-dependent differences in the relationship between EEG signals and spiking activity in V1.

As shown in figure 9, MUAe predictions at superficial layers were generally better than deep layers, particularly for flickering stimuli. For trial-averaged signals, all flickering conditions showed significantly better model performance at shallow than deep layers (5 Hz, Δ*r* = 0.063, *p* = 0.0003; 10 Hz, Δ*r* = 0.049, *p* = 0.0026; 20 Hz, Δ*r* = 0.036, *p* = 0.017; 40 Hz, Δ*r* = 0.027, *p* = 0.052), which was not seen for spontaneous 0 Hz condition (Δ*r* = −0.002, *p* = 0.55). A similar trend was observed for single-trial signals for both flickering and spontaneous conditions (0 Hz, Δ*r* = 0.000, *p* = 0.49; 5 Hz, Δ*r* = 0.002, *p* = 0.079; 10 Hz, Δ*r* = 0.003, *p* = 0.0051; 20 Hz, Δ*r* = 0.006, *p* = 3.2 × 10^−6^; 40 Hz, Δ*r* = 0.004, *p* = 0.0019). These results suggest that EEG predicts spiking activity more accurately in the superficial layers, supporting the idea that superficial layers contribute more to the EEG signal than deeper layers.

## 4. Discussion

### 4.1. Summary

Neuronal spiking activity is a local signal reflecting both stimulus and cognitive information dynamically at millisecond resolutions, making it a primary focus of investigation in neuroscience. However, given the need for invasive methods, this signal is typically studied in animals. In contrast, EEG, which is non-invasive and typically recorded in humans, offers sub-second temporal resolution but is constrained by poor SNR and spatial resolution. The relationship between these two signals is not well established, limiting the potential to estimate neural dynamics non-invasively with high temporal precision.

With the high-level goal of estimating temporally precise neural activity non-invasively, this study aimed to predict V1 spiking activity (MUAe) from EEG recordings in a behaving macaque. We implemented two major methodological advancements: (1) using flickering stimuli to elicit high-SNR SSVEP responses in EEG, and (2) developing a comprehensive model to predict V1 MUAe using spectrotemporal features of EEG signal. We found that V1 spiking activity could be predicted from EEG, particularly during trials with flickering stimuli, both at the trial-averaged and single-trial levels. Interestingly, model performance improved further when the phase of the flickering stimulus frequency was included as an additional predictor. Subsequent analysis showed that EEG-to-MUAe prediction accuracy was higher for superficial than deep cortical layers.

### 4.2. Limitations

Although we recorded from multiple cortical (*n* = 32) and EEG (*n* = 3) electrodes across 14 sessions, with an average of 308 trials per session, our study relies on data from a single monkey, which limits the interpretation of our findings in several ways. First, we could not empirically verify whether the results replicate in a second monkey using a similar recording setup, a standard approach in non-human primate electrophysiology research. Second, electrode positions were fixed within V1. Because neurons in V1 exhibit temporal-frequency tuning (Foster *et al* 1985, Zheng *et al* 2007, Yu *et al* 2010), the stimulus frequency-dependent effects—such as the stronger EEG-MUAe relationship for 10 Hz compared to other stimulus conditions—may be influenced by the specific, idiosyncratic placement of electrodes and may not generalize across the entire V1. Therefore, in the discussion, we focus primarily on findings less likely to be affected by electrode placement. Specifically, we discuss the differences between flickering and non-flickering stimuli, as well as the effects of model parameters independent of stimulus frequency. In addition, because our prediction model (equation (1)) is designed to estimate the spiking activity envelope (MUAe), which reflects the aggregated spiking activity of multiple local neurons, its performance may be suboptimal for predicting single-unit spiking activity. Although the model performs well for our current behavioral paradigm and, due to its comprehensive nature, is likely applicable to other paradigms as well, we have not empirically tested this. Therefore, the question of its generalizability across tasks and brain regions remains open for future studies.

### 4.3. EEG and spiking activity are related in a frequency-dependent manner

Studies investigating simultaneously recorded EEG and spikes in anesthetized animals date back to the 1960s. Earlier studies involving invasive recordings from the epidural surface and spiking signals from the visual cortex in anesthetized animals showed that the majority of recorded cells fire faster during positive surface potential and fire less during negative surface potentials, both under spontaneous and visual flash stimulus conditions (Fromm and Bond 1964, Fromm and William Bond 1967). A series of studies by Schroeder and colleagues reported a detailed investigation of visually evoked potentials recorded from the epidural surface and underlying spiking/LFP signals in anaesthetized macaques. They showed that the spiking activity of different visual processing areas contributes to different components of surface potentials. For example, early components (<80 ms) of evoked potentials on the cortical surface indicate V1 processing and late components (100–200 ms) indicate visual processing from extrastriate areas, such as V4 (Schroeder *et al* 1991, Givre *et al* 1994).

A few studies in the last two decades investigated a more detailed EEG-spike relationship using less- or non-invasive methods in behaving animals. The direct correlation between non-invasive scalp EEG and spiking activity in V4 was observed to be very weak in macaque monkeys (Snyder and Smith 2015). However, EEG measured on the bone surface showed a clearer relationship with V1 spiking activity (Whittingstall and Logothetis 2009). Particularly, the EEG-spike relationship was observed to be frequency dependent during naturalistic visual stimulus, where the phase of low-frequency (delta/theta) and amplitude of high-frequency (gamma) bands were better related to V1 spikes than mid-frequency bands (alpha/beta). This frequency-specific EEG to V1 spike relationship also extends to LFPs (Rasch *et al* 2008).

We extend these earlier findings to non-invasive EEG, particularly by evaluating the relationship between V1 spiking signal and non-invasive EEG in behaving macaques. Similar to previous LFP and invasive EEG findings, we found that EEG frequency bands are differentially related to V1 spiking activity. For the spontaneous condition (0 Hz stimulus) at a single-trial level, which can directly be compared with previous studies (Rasch *et al* 2008, Whittingstall and Logothetis 2009), we found that delta and gamma bands of EEG were correlated with MUAe (supplementary figure 2). However, we also observed that the alpha band shows a negative correlation, which was not observed in previous studies. We attribute this to differences in the animal state and the visual stimuli. A simple fixation task with no varying stimulus can be less engaging than the naturalistic movie-viewing paradigm used in previous studies (Rasch *et al* 2008, Whittingstall and Logothetis 2009), thereby affecting alpha amplitude and its correlation with spikes (Klimesch 1999).

### 4.4. EEG-spike relationship improves for visual stimulus eliciting steady-state responses

Repetitive visual stimuli elicit steady-state responses in the visual cortex, characterized by high SNR (Norcia *et al* 2015). Due to high SNR, these responses can easily be observed in non-invasive EEG, making them a popular choice in human neuroscience. We leveraged high-SNR responses of repetitive visual stimulus to relate spiking activity in V1 with non-invasive EEG. As expected, SSVEP responses were readily detectable in monkey EEG, and the EEG-spike correlation was stronger for all flickering stimulus conditions than for the non-flickering condition (figures 3 and 4(a)). Furthermore, when comparing among flickering conditions, we observed that the EEG-spike relationship depended on the stimulus frequency (figures 3 and 4(a)).

These results support the idea that visual stimuli influence the EEG-spike relationship (Snyder and Smith 2015), and help extend and interpret the previous observations made by Whittingstall and Logothetis (2009). They showed that delta and gamma frequency bands are better correlated with spiking activity in monkeys engaged in a naturalistic movie-viewing task, similar to our findings for single-trial spontaneous conditions (supplementary figure 2). However, their observed delta-gamma relationship with spikes may be specific to the naturalistic movie paradigm, which elicits visual responses in the delta frequency range (3–4 Hz), as discussed in detail by Mazzoni *et al* (2010). Our experiments extend this beyond delta-gamma coupling by showing that several phase-amplitude relationships of the EEG signal are informative about spiking activity and that this depends on the frequency of the visual stimulus.

We speculate that the entrainment of cortical neurons to an external rhythm enhances their synchronous firing (Lakatos *et al* 2019, Huang *et al* 2021), which likely contributes to the EEG signal (Murakami and Okada 2006, Musall *et al* 2014, Snyder *et al* 2015), and thereby renders it more predictable than the non-synchronized activity observed in the absence of periodic visual input.

### 4.5. Mechanisms underlying EEG predictors of MUAe

In our model, amplitude, phase, and coupling predictors all contributed to performance (figure 7), with phase and PAC proxies (interaction terms) outperforming amplitude features, especially during flicker conditions. Relating individual EEG predictors to the underlying cortical mechanisms is nontrivial, and defining circuit-level mechanisms is beyond the scope of this study. Still, previous studies offer a plausible interpretive framework.

Biophysical modeling studies show that multiple factors, such as dendritic geometry, postsynaptic currents, and the synchrony of population activity, shape EEG signals at the scalp (Murakami and Okada 2006, Thio and Grill 2023). It is thought that postsynaptic currents contribute the majority of the signal, but contributions from spikes are not negligible (Buzsáki *et al* 2012, Thio and Grill 2023). This suggests that amplitude features (particularly high-frequency power) likely capture variations in synaptic drive that correlate with firing rate, or synchronous spiking activity, so increases in gamma/broadband power provide a proxy for elevated spiking (Rasch *et al* 2008, Ray *et al* 2008, Ray and Maunsell 2011).

The phase of slower rhythms likely indexes temporal windows of cortical excitability, so phase predictors capture when neural populations are more permissive of firing (Lakatos *et al* 2005, Schroeder and Lakatos 2009). With phase as an input, the model can use this relationship to predict spiking activity. Using multiple bands, each with its own oscillatory period, provides this advantage at multiple time scales; slower bands index sparsely repeating permissive windows (predicting changes over longer timescales), and faster bands index shorter, more frequent permissive windows (predicting spikes with finer temporal precision).

The interaction terms in the model likely rely on the neural mechanisms of PAC, which measures the statistical dependence between low-frequency phase and high-frequency amplitude (Tort *et al* 2008, Canolty and Knight 2010). Statistically, the interaction terms benefit from the synergy of combining two relatively weak sources of information (amplitude and phase). For instance, gamma power at some time point may, on its own, be too low to infer strong spiking activity, and a delta phase permissive of spiking is also, on its own, not sufficient to reliably infer spiking activity. But when both gamma power and a permissive delta phase are observed simultaneously, they can be used to infer higher spiking activity with higher confidence.

### 4.6. Phase of stimulus frequency further improves EEG to spike predictions

Mazzoni *et al*’s biophysically realistic model suggested that periodic stimulation in the LGN entrains V1 activity to the same rhythm, thereby influencing the EEG-spike relationship (Mazzoni *et al* 2010). They hypothesized that low-frequency fluctuations (delta/theta) reflect shifts in cortical excitability via the modulation of local excitatory-inhibitory (E-I) loops, which results in high-frequency activity (gamma). This combination of distinct information in low- and high-frequency bands can be used to estimate local spiking activity. Notably, Mazzoni *et al* (2010) proposed that if slow fluctuations in V1 are induced externally (e.g. through LGN stimulation), this rhythm could enhance EEG-to-spike estimations.

To test this hypothesis, we induced rhythmic activity in V1 using visual flicker at a specific frequency rather than directly stimulating the LGN through electrical or optical methods. We based our approach on the established fact that visual information from the retina is primarily relayed to the LGN and subsequently to V1; LFP spectra in our data also show that V1 responses followed the visual flickering rhythm (not shown), similar to V1 responses for periodic optogenetic stimulation of the dorsal LGN (Perrenoud *et al* 2025). Consistent with Mazzoni *et al*’s hypothesis, we found that our model incorporating the stimulus phase outperformed the model without it, particularly so for single-trial estimations.

### 4.7. EEG is better related to spikes at the superficial than deep cortical layers

The neocortex is organized into layers, each characterized by unique cytoarchitectural features. We investigated whether EEG-to-spike predictions differ across cortical layers and found that MUAe at electrode sites in shallow layers are better predicted than in deep layers (figure 9). In general, worse predictions with increasing depth are consistent with the expected smearing effects of volume conduction (Subramanian *et al* 2025). However, the relatively small distance (∼500 *μ*m) between the shallow and deep layers within a cortical column suggests that volume conduction alone cannot fully explain this finding (Thio and Grill 2023). We suspect that the stereotypical variation in the LFP power in different frequency bands across cortical layers is a contributing factor. Notably, gamma power is stronger in the LFP of superficial layers, while alpha and beta power are stronger in deeper layers (Mendoza-Halliday *et al* 2024). Since gamma activity is closely associated with spiking activity in the visual cortex (Ray *et al* 2008, Ray and Maunsell 2011), our findings support the hypothesis that this gamma power, when recorded from the scalp with EEG, results in better prediction of spikes in superficial layers.

### 4.8. Implications for human EEG and non-invasive BMI

In this non-human primate study, we employed non-invasive EEG, the standard method for recording EEG in humans. This approach facilitates translation of our findings in human EEG and is especially valuable for advancing EEG-based non-invasive BMI. Under spontaneous conditions, the correlation between EEG and spiking activity is weak (figures 2, 3 and 4(a) and see also Snyder and Smith 2015), making spike predictions from EEG challenging. However, as discussed above, incorporating a broader array of EEG spectrotemporal features and SSVEP stimuli can improve this accuracy. Based on these improvements, our study offers three insights that are directly applicable to human EEG, paving the way for more effective non-invasive decoding of spike dynamics and associated behaviors.

First, given that EEG frequency bands are differentially related to spike dynamics, we recommend utilizing band-limited EEG rather than full-band EEG for spiking and behavioral decoding. Second, the model shows that amplitude, phase, and PAC variables of EEG bands are all informative in predicting spiking signals (figure 7). Interestingly, phase and coupling variables were more informative than amplitude variables, particularly for single-trial estimations, indicating the potential use of phase and coupling variables in real-time BMI applications. Third, our observations that visual flicker eliciting SSVEP responses improves EEG-to-spike predictions suggests that using stimuli with periodic fluctuations may be a valuable approach to improve prediction accuracy. This strategy could be leveraged in other sensory modalities and highlights the potential of stimulus-driven rhythmic stimulation to optimize neural decoding. In addition, incorporating both amplitude- and phase-related EEG features into more flexible, computationally intensive nonlinear models could further improve EEG-to-spike predictions (Hu *et al* 2022).

The improvement in EEG-to-spike predictions achieved through periodic flickering stimuli also suggests the potential for using frequency-tagging methods to investigate various cognitive functions non-invasively. Frequency tagging involves associating task-relevant cues with non-task-relevant flickering stimuli to elicit high SNR SSVEP responses in EEG (Vialatte *et al* 2010, Zhu *et al* 2010, Norcia *et al* 2015). Our findings suggest that analyzing the spectrotemporal features of EEG responses to frequency-tagged stimuli could result in a deeper understanding of the neural basis of complex cognitive functions in a non-invasive manner (Peterson *et al* 2014, Zhigalov *et al* 2019, Arora *et al* 2024, Ladouce and Dehais 2024). Future studies are needed to test this hypothesis.

## Data Availability

The code for data analysis and the data that support the findings of this study are openly available at the following URL/DOI: https://osf.io/vq5th. Supplementary Figures available at https://doi.org/10.1088/1741-2552/ae2541/data1.
